# Sensing oxygen inside and out

**DOI:** 10.7554/eLife.27467

**Published:** 2017-05-19

**Authors:** Maria R Stupnikov, Wellington V Cardoso

**Affiliations:** 1 Columbia Center for Human Development, Department of Medicine, the Division of Pulmonary, Allergy and Critical Care Medicine, and the Department of Genetics and Development, Columbia University Medical Center, New York, United States; 1 Columbia Center for Human Development, Department of Medicine, the Division of Pulmonary, Allergy and Critical Care Medicine, and the Department of Genetics and Development, Columbia University Medical Center, New York, United Stateswvc2104@columbia.edu

**Keywords:** sea lamprey (Petromyzon marinus), neural crest, endoderm, neuroepithelial cells, carotid body, fate-mapping, Chicken, Mouse, *Xenopus*, Zebrafish, Other

## Abstract

Neuroendocrine cells act as oxygen sensors in animals from fish to humans, but the evolutionary origins of these cells are only just becoming clear.

**Related research article** Hockman D, Burns AJ, Schlosser G, Gates KP, Jevans B, Mongera A, Fisher S, Unlu G, Knapik EW, Kaufman CK, Mosimann C, Zon LI, Lancman JJ, Dong PDS, Lickert H, Tucker AS, Baker CVH. 2017. Evolution of the hypoxia-sensitive cells involved in amniote respiratory reflexes. *eLife* **6**:e21231. doi: 10.7554/eLife.21231

For single-celled organisms like bacteria, every cell can detect changes in the level of oxygen in its environment and respond accordingly. Things, however, can be rather more complicated in multicellular organisms (Jonz et al., 2016). For example, animals must have a respiratory system to deliver enough oxygen to millions of cells to meet with their metabolic demands. An animal’s survival also depends on it monitoring oxygen levels both internally (for example, in its blood) and externally (in the environment). Multicellular organisms have oxygen sensors that consist largely of cells called neuroendocrine cells. When oxygen levels drop, these sensors release neurotransmitters, chemicals that excite nearby neurons to signal to a control center in the brain. The control center then triggers reflexes that increase the frequency of breathing.

These oxygen sensors have evolved at strategic places in the body. In land animals like mammals and birds (collectively referred to as amniotes), these sensors are found in two distinct locations: next to the carotid arteries, and in the airways of the lungs. The carotid arteries are the major blood vessels that deliver oxygenated blood to the head and neck, and the neuroendocrine cells are packed closely together to form a structure called the carotid body located where these arteries branch. In the airways of the lungs, the oxygen sensors are distributed as single cells or in small groups known as neuroendocrine bodies, present mostly at the branch-points. Primarily aquatic species, like fish and amphibians (collectively referred to as non-amniotes), have oxygen-sensing neuroendocrine cells in their gills to detect oxygen in the water. At least one fish, the jawless lamprey, also has oxygen-sensitive cells that are rich in neurotransmitters near its major blood vessels, reminiscent of the carotid body found in the land animals (Jonz et al., 2016).

The multiple similarities of neuroendocrine cells in these different structures have led to a long-lasting debate as to how they are related in terms of evolution, particularly whether the neuroendocrine cells in the carotid body and the lungs of land animals evolved from the neuroendocrine cells in the gills of their aquatic ancestors. Now, in eLife, Clare Baker at the University of Cambridge and colleagues – including Dorit Hockman as first author – address this controversy and report that these different oxygen sensors diverged long before animals transitioned onto land (Hockman et al., 2017).

Previous studies in mice and birds had already shown that lung neuroendocrine cells have a different embryonic origin than those found in the carotid body. Most animal embryos form three distinct layers during the early stages of development: endoderm, mesoderm and ectoderm. These layers later contribute to different parts of the body. The neuroendocrine cells in the lung develop from the endoderm, while those in the carotid body develop from the neural crest, which arises from the ectoderm (Pearse et al., 1973; Hoyt et al., 1990; Song et al., 2012; Kuo and Krasnow, 2015).

To decipher the evolutionary relationships between the oxygen sensors, Hockman et al. – who are based at institutions in Germany, Ireland, the Netherlands, the US and the UK – set out to determine the origin of the gill neuroendocrine cells. To do this, they traced how neuroendocrine cells develop in the embryos of three non-amniotes: namely two species of fish (lamprey and zebrafish) and one species of frog (*Xenopus*). First, they engineered zebrafish so that all cells derived from the neural crest would fluoresce red. However, no red cells were seen in the gills, which indicated that gill neuroendocrine cells are not neural crest-derived. The finding that zebrafish mutants which lack neural crest cells still developed neuroendocrine cells in their gills further supported this conclusion. Importantly, Hockman et al. went on to confirm that the neuroendocrine cells in the zebrafish gills develop from endodermal cells, just like those in the lungs of mice. Similar results were seen with lamprey and *Xenopus*.

The finding that during development neuroendocrine cells in gills arise from the endoderm and not the neural crest effectively ruled out the possibility that they could have evolved from the same precursor cell as carotid body cells. Hockman et al. then identified neural crest-derived cells rich in neurotransmitters near major blood vessels in young lamprey and zebrafish. Specifically, these cells were found near large blood vessels of the fish’s pharyngeal arches, an association that closely resembles that seen in the carotid bodies of land animals. This led Hockman et al. to propose a new model for the evolution of oxygen sensors (Figure 1). According to this model, the carotid body seen in amniotes evolved from the grouping together of the neural crest-derived cells that were present near the pharyngeal arches of non-amniotes (which eventually develop into the gill arches). Later, these cells altered the neurotransmitters that they produced, and became wired into the nervous system. By contrast, the neuroendocrine cells in the gills and lungs continued to develop in situ from endodermal cells when animals transitioned from an aquatic to a terrestrial life.

**Figure 1.**
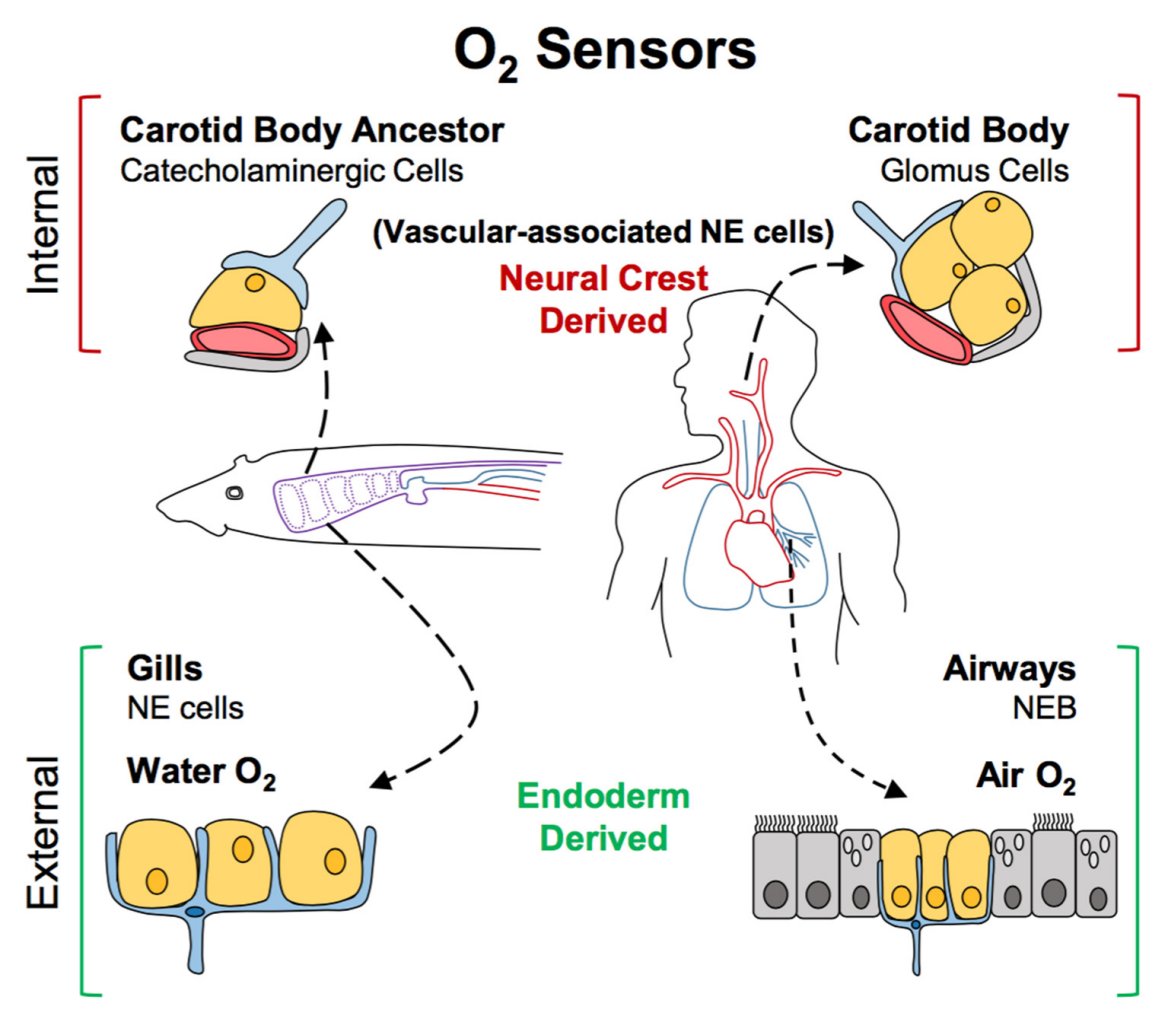
A new model for the evolution of oxygen sensors. Oxygen-sensitive neuroendocrine (NE) cells (yellow) associated with blood vessels (red) serve as sensors for internal oxygen levels (top). These include catecholaminergic cells in an ancestral structure in non-amniotes like fish (left), and the glomus cells in the carotid body of amniotes like humans (right). Other neuroendocrine cells act as sensors for external oxygen (bottom). These include cells in the gills of fish (left) and the airways of amniotes (right). Hockman et al. propose that the clusters of catecholaminergic cells near the blood vessels in non-amniotes evolved into the carotid bodies of amniotes. The neuroendocrine cells in these internal sensors are all derived from the neural crest. By contrast, the external oxygen sensors in gills and airways are derived from the endoderm in both non-amniotes and amniotes. Neurons are shown in blue; accessory cells are shown in gray. NEB: neuroendocrine body.

The challenge ahead is to elucidate the network of genes that led oxygen-sensing structures to diversify as animals evolved. Some of the signals needed for neuroendocrine cells to develop are already known (Borges et al., 1997; Ito et al., 2000; Kameda, 2005; Tsao et al., 2009; Branchfield et al., 2016). However, there is evidence that each type of sensor requires slightly different cues and produces a different combination of molecules (Dauger et al., 2003). Additional knowledge of these differences will help us to understand how these sensors acquired their specialized functions to fulfill new roles during evolution.
